# Adsorption Performances and Mechanisms of MgFe_2_O_4_ Spinel Toward Gallium (III) from Aqueous Solution

**DOI:** 10.3390/ma17235740

**Published:** 2024-11-23

**Authors:** Loredana Ciocărlie, Adina Negrea, Mihaela Ciopec, Narcis Duteanu, Petru Negrea, Paula Svera (m Ianăși), Cătălin Ianăşi

**Affiliations:** 1 Faculty of Chemical Engibeering, Biotechnologies and Environmental Protection, Polytechnic University of Timişoara, Victoriei Square, No. 2, 300006 Timisoara, Romania; 2 National Institute for Research and Development in Electrochemistry and Condensed Matter, 144th Dr. A.P. Podeanu Street, 300569 Timisoara, Romania; paulasvera@gmail.com; 3 National R and D Institute for Welding and Material Testing ISIM Timisoara, 30 Mihai Viteazul Blv, 300222 Timisoara, Romania; 4 “Coriolan Drăgulescu” Institute of Chemistry, Bv. Mihai Viteazul, No. 24, 300223 Timisoara, Romania; ianasic@acad-icht.tm.edu.ro

**Keywords:** sol–gel synthesis, gallium recovery, kinetics study, isotherms, adsorption, MgFe_2_O_4_ spinel

## Abstract

The European Union regards gallium as a crucial element. Because of that, the retrieval of gallium ions from secondary sources through diverse methodologies is of the utmost significance in an actual economical context. The primary goal of this study was to explore the viability of MgFe_2_O_4_ spinel as an adsorbent material for Ga(III) ions recovery from aqueous solutions. A spinel adsorbent material was synthesised by using the sol–gel synthesis method. After preparation, the obtained spinel was subjected to a thermal treatment, which resulted in modifications of its crystalline structure and morphology, in concordance with the calcination temperatures. Specifically, two distinct temperatures of 260 and 650 °C were utilised in the process, which was conducted in air. The second objective was represented by the physicochemical characterisation of the newly prepared adsorbent material by using various analytical techniques, e.g., Fourier transform infrared spectroscopy (FT-IR), atomic force microscopy (AFM) and magnetic measurements. The optimal conditions for Ga(III) adsorption were established (S:L ratio, solution pH, contact time, temperature, initial Ga(III) concentration). Simultaneously, the obtained experimental data were modelled to prove the fact that the pseudo-second-order model explained the studied kinetics process and established its mechanism. Intraparticle diffusion was also studied to highlight the rate-determined step during the Ga(III) adsorption process. The equilibrium of the process was also studied, establishing that the Sips isotherm fitted the experimental data best, with a correlation coefficient R^2^~1, indicating that the studied adsorption process was homogeneous, the maximum adsorption capacity of spinel being 24.7 mg Ga (III)/g MgFe_2_O_4_. Thermodynamic parameters, involving ΔG°, ΔH° and ΔS°, were also calculated; negative values of ΔG° indicated that the adsorption was spontaneous. ΔH° proved to be endothermic, and the calculated ΔS° values being positive confirmed the fact that the process was spontaneous.

## 1. Introduction

In nature, gallium is not found in its elemental form, but it can be found in the form of gallium salt (III), especially in zinc ores or bauxite. Gallium is a silvery metal with a tannic taste and a glassy odourless surface. It is solid, soft and brittle, and, when broken, it forms conchoidal fragments, which are curved and resemble seashells. When the gallium melts, if we look at it from a specific angle, we can see a bluish glow. It is not toxic on contact but sticks very easily to surfaces (mainly ceramics/glass). Solid-state devices and semiconductor manufacturing employ gallium usage. Similarly, coherent light production by electricity conversion is possible by using gallium arsenide. Another example is represented by commercial UV-activated powder phosphors, which are produced using magnesium gallate containing divalent impurities like Mn^2+^. Traces of gallium can be found in bauxite, germanite, sphalerite, coal and diaspore. For example, the gallium concentration in coal combustion dust can reach 1.5%. Pure metallic gallium can be obtained by electrolysing its hydroxide in a KOH solution [1,2]. Gallium is a rare low-melting-point metal that mixes easily with most elements to form low-melting-point alloys. This characteristic makes gallium and its compounds widely utilised in both industrial and medical domains. As a particular example, in nuclear medicine, radioactive gallium is used to detect and localise cancerous tumour cells. Gallium’s unique semiconductor properties, which enable the transformation of electrical energy in light, and gallium compounds, including gallium nitride, are often used in the construction of electrical devices such as light-emitting diodes (LEDs) [3,4,5,6]. In the actual development stage, gallium compounds gain a lot of interest because of their superior semiconducting properties. Gallium arsenide and gallium nitride are widely used in the manufacturing process of various gadgets, such as DVD laser diodes, and in different other industrial and electronic items.

Different high-tech industries, including infrared optics, fibre optic systems, detectors and semiconductor devices (transistors, diodes and rectifiers), are dependent on gallium. In this context, gallium is predicted to become considerably higher in demand due to the rapid expansion of high-tech industries; therefore, there is a lot of interest in using all gallium resources [7,8].

In real raw materials, gallium rarely exists in its concentrated form; however, gallium is found only in association with aluminium in bauxite and zinc in numerous other minerals. In this context, gallium can be recovered as a by-product during zinc and aluminium production. Gallium, which is found in zinc minerals, is rarely recovered due to the limitations associated with its extraction in different acidic systems. In this situation, the largest quantities of gallium are obtained as by-products from the hydrometallurgical processing of bauxite. The relative saturation of aluminium production correlated with the increasing demands for gallium make its recovery from zinc minerals an important objective. At this time, 80% of zinc used worldwide is produced by hydrometallurgical technologies, where the obtained residues end up being enriched with gallium [4,5,6].

Over time, several different methods for gallium recovery have been developed, especially Bayer liquor resulting from alumina processing [7], fractional precipitation (lime and, respectively, carbonation method), electrochemical recovery methods, cementation [9] and solvent and ion exchange. When gallium is recovered by using fractional precipitation technology, lime or CO_2_ are introduced into the system, ensuring the further coprecipitation of the gallium oxide concomitant with alumina. In the second step, Al and Ga are separated from the mixture by treatment with NaOH solutions, where Al and Ga hydroxides are dissolved. Metallic gallium is recovered from this solution by carbonisation [10].

Keeping in mind the necessity to preserve the environment, cleaning the wastewaters before releasing them is vital. So, in this context, it is important to have a proper technology for this treatment [11,12]. Gallium recovery from Bayer liquor by using solvent extraction has been extensively investigated during the past two decades, and usually Kelex 100 was used as the solvent because this compound presents a good ability to extract gallium from aluminate solutions [13]. Nevertheless, this method presents several disadvantages, e.g., relative low extraction rate, concomitant with high operational costs and oversensitivity of different external parameters. Further experiments have proven that the ion exchange method represents the most efficient process used for gallium recovery, being applied in almost all industrial practices due to is fast kinetics, high recovery efficiency and good selectivity for gallium recovery [7]. Another method used for gallium recovery from aqueous solutions is represented by adsorption. In this process, the adsorbent material used for gallium recovery presents a high importance. Screening the literature data, it was found that different materials are used as adsorbents for gallium recovery, such as mesoporous activated carbons [14], ion exchangers [15], biomaterials [16] or zeolites [17]. A different technology used to recover Ga ions from aqueous solutions involves the preparation of ion-imprinted bio-adsorbent materials, such as persimmon peel-base imprinted adsorbent material [18]. Some studies demonstrate that Cu/Mg spinel composites present a high efficiency for removal of different colourants [19]. Recent studies show that spinel MgFe_2_O_4_ has significant adsorption capacities, especially in the case of removing dyes from water [20,21]. However, only a couple of studies have been conducted on heavy metal adsorption from water [22].

Magnesium ferrites represent a versatile material presenting an AB_2_O_4_ crystalline structure, where the oxygen atoms play an important role in crystalline network formation. Temperature increase induces some changes in the crystalline network when tetrahedral or octahedral structures are formed. In the case of the magnesium ferrites, magnesium ions occupy the meshes of the octahedral network and iron ions occupy the meshes of the tetrahedral network. In 2021, McDonald et al. proved that magnesium ferrite presents a much higher stability when it crystallises in a mixed inverse conformation [23]. MgFe_2_O_4_ spinel can be prepared by using different synthesis methods, such as solid-state [24], combustion [25], hydrothermal [26], sol–gel [27], reverse microemulsion route [28], sol–gel synthesis using the Pechini route [29], single-step synthesis via the combustion route [30], sol–gel/combustion method [27,31], hydrothermal decomposition of double-layered double hydroxide (obtained by coprecipitation of magnesium and iron hydroxides) [32,33], hydrothermal synthesis in supercritical water [34], thermal synthesis from MgO and Fe_2_O_3_ [35], solvothermal method followed by subsequent sintering [36] and coprecipitation [37,38,39]. Due to its properties, MgFe_2_O_4_ spinel has different industrial applications in various electrical [40], photocatalytic [41] and magnetic applications, [23,40] and also as an adsorbent material [22]. In actual technological context, gallium recovery from secondary sources represents a main issue. Therefore, in the present paper, we investigate the preparation and subsequent utilisation of magnesium ferrites as an adsorbent material for gallium recovery from aqueous solutions.

## 2. Materials and Methods

The desired adsorbent material was prepared via the combustion method, as reported in our previous paper [38]. After preparation, the synthesised material was characterised by using the analytical techniques presented in detail in the following paragraphs. After physicochemical characterisation, the newly prepared material was tested as an adsorbent material for Ga(III) recovery in order to prove its ability to retain Ga(III) ions and to determine the optimum conditions for the studied adsorptive process.

### 2.1. Material Synthesis and Characterisation

#### 2.1.1. MgFe_2_O_4_ Composite Synthesis

**Figure 1:**
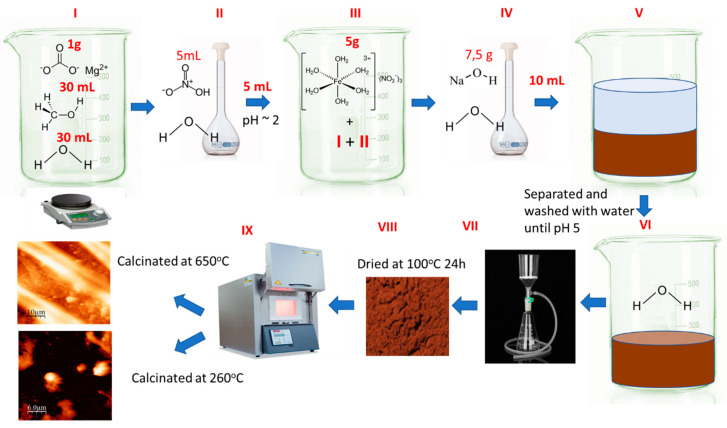
Schematic of the MgFe_2_O_4_ composite synthesis.

MgFe_2_O_4_ spinel was synthesised by the coprecipitation method using magnesium carbonate as the magnesium source [38]. During preparations, 1 g of magnesium carbonate (SC Chimopar Trading SRL, Bucharest, Romania) was used, which was mixed with 30 mL of DI water and 30 mL of methanol (SC Chimopar Trading SRL, Bucharest, Romania). Then, into the obtained solution, 5 mL of nitric acid (SC Chimopar Trading SRL, Bucharest, Romania) was added in order to allow carbonate complete dissolution and to regulate the pH at 1.5–2. The obtained solution was mixed for 1 h, followed by the addition of iron (III) nitrate (SC Chimopar Trading SRL, Bucharest, Romania). This new solution was kept at 50 °C under continuous stirring for another 3 h. Precipitation of the desired compound was achieved by the addition of a sodium hydroxide solution in order to obtain a final pH of 5. In the final stage, the prepared material was cleaned with DI water to remove any remaining sodium hydroxide, then was dried at 100 °C for 24 h. Then, the dried material was calcined at temperatures of 260 and 650 °C using a temperature increase rate of 5° per minute. A schematic representation of the adsorbent material preparation is presented in Figure 1.

#### 2.1.2. MgFe_2_O_4_ Composite Physicochemical Characterisation

##### Fourier Transform Infrared Spectroscopy (FT-IR) Analysis

After the adsorbent material preparation, Fourier transform infrared spectroscopy (all spectra were recorded using ASCO FT/IR-4200, Shimadzu, Japan) was used to characterise the adsorbent materials obtained after crude material calcination. This technique was used in order to confirm the preparation of the desired MgFe_2_O_4_ adsorbent material.

##### Magnetic Measurements

Magnetisation curves were recorded by using a homemade induction magnetometer [42], involving an AC field (50 Hz) with 5 kOe amplitude. During these tests, Ni powder (99.8% purity, 30 μm mean grain diameter, Sigma-Aldrich) was used as a reference for calibration. MgFe_2_O_4_ spinel magnetisation was obtained by extrapolation of the recorded data with a field of 5 kOe.

##### Atomic Force Microscopy (AFM) Analysis

The adsorbent materials obtained after synthesis and thermal treatment were analysed by atomic force microscopy (AFM). AFM images were recorded by using a scanning probe microscopy platform (MultiView-2000 system, Nanonics Imaging Ltd., Jerusalem, Israel) in intermittent mode at 298 K. In order to carry out the AFM analysis, a chromium-doped tip with a 20 nm radius and 30–40 kHz resonance was used. 

### 2.2. Ga(III) Adsorption Experiments

A number of parameters that affected the studied adsorptive process on MgFe_2_O_4_ (S:L ratio, pH, duration, Ga(III) initial concentration and temperature) were examined for a better understanding of the mechanism associated with Ga(III) recovery on synthesised adsorptive materials.

#### 2.2.1. Solid/Liquid (S:L) Ratio Effect

In order to understand how the S:L ratio influenced the gallium recovery from different liquid solutions, several experiments were performed where the amount of adsorbent material was varied. The Ga(III) solution volume, with an initial concentration of 100 µg Ga L^−1^ (prepared form a stock solution Ga(NO_3_)_3_ in HNO_3_ 2–3%, Merck, Darmstadt, Germany, 1000 mg L^−1^ Ga Certipur), was kept constant in all experiments. Similarly, all adsorptive processes were conducted at a constant temperature of 298 K. Thus, in 0.025 L of solution, different adsorbent material ratios between 0.025 g and 0.3 g of MgFe_2_O_4_ were added. All samples were shacked in a thermostatic bath (Julabo SW 23, JUlabo GmbH, Seelbach, Germany) for 60 min at a constant temperature of 298 K.

#### 2.2.2. Influence of the pH Value on Ga(III) Adsorption Process

Another crucial parameter for all adsorptive processes was the pH of the aqueous solution. To evidence the impact of the pH solution on G(III) adsorption, experiments were carried out by using solutions with pHs between 1 and 10. In all these cases, the initial concentration of the Ga(III) solution was 100 µg L^−1^. All adsorptive experiments were performed by weighting samples of 0.1 g of adsorbent material, which were mixed with gallium solutions (having different pHs) and stirred for 1 h at a temperature of 298 K. In order to adjust solution pHs at desired values, HNO_3_ solutions (having concentrations from 0.05 M to 2 M HNO_3_) or NaOH solutions with similar concentrations were used. Furthermore, solution pHs were recorded by using a pH meter produced by METTLER TOLEDO (Mettler Toledo SevenCompact pH meter, Columbus, OH, USA).

#### 2.2.3. Influence of Contact Time and Temperature

Two additional important parameters for all adsorptive processes are represented by contact time and temperature. The effects of these two parameters on the studied adsorptive process can affect the compatibility of the prepared adsorbent material for Ga(III) ions recovery. Therefore, these two parameters can be a limitation factor for gallium recovery. In this context, after 0.1 g of adsorbent material was weighed, 0.025 L of Ga(III) solution having an initial concentration of 100 µg L^−1^ was added in order to set out the impact of temperature and contact time on the studied adsorptive process. The samples were agitated at 200 rpm for different time periods (15–120 min) using a thermostatic water bath at four different temperatures (298, 308, 318 and 328 K) at 200 rpm.

#### 2.2.4. Influence of Initial Concentration

The maximum adsorption capacity of the newly prepared adsorbent material was determined by preparing different Ga(III) solutions with concentrations ranging from 100 to 140,000 µg L^−1^, having the pH equal the pH determined in the previous experimental step. Also, during these experiments, the optimum contact time and temperatures evaluated during earlier tests were used. After the adsorptive experiments were performed, samples were filtered and the Ga(III) residual concentration was measured by atomic adsorption spectrometry (using an atomic adsorption spectrometer equipped with graphite furnace, AA 6800, Shimadzu, Japan).

The adsorbent material adsorption capacity, q (µg g^−1^), was evaluated using the following equation:

$$q=\frac{\left(C_{0}-C_{f}\right)V}{m}$$

where C_0_ is the initial concentration of Ga ions in aqueous solution, (µg L^−1^);

C_f_ is the residual concentration of Ga (III) in the solution, (µg L^−1^);

V is the solution volume, (L);

m is the mass of adsorbent, (g).

#### 2.2.5. Evaluation of the Absorption Process Mechanism

Adsorption, kinetics, thermodynamic and equilibrium studies were carried out in order to elucidate the mechanism associated with the Ga(III) adsorption process.

##### Kinetics Studies

The behaviour of any adsorbent material from a kinetic and thermodynamic point of view determines the efficiency of the adsorption process. Any adsorbent material with a good adsorption capacity but a relatively low reaction speed is not a viable alternative, due to the fact that it requires a longer time for the adsorbed molecules to reach active centres located inside the adsorbent particles.

On the contrary, an adsorbent characterised by a high reactivity but a low adsorption capacity is not a desired one because the adsorption process will require a large quantity of adsorbent material, which would lead to an increase in additional costs. The ideal material for the adsorption process is an adsorbent that presents a high adsorption capacity and a high reaction rate [43]. Information regarding the ideal conditions, the adsorption mechanism and the reaction speed of the investigated adsorptive processes (which are able to prove whether the studied process is a mass transfer process or a chemical reaction) are obtained by analysing the data obtained from the kinetics studies.

Kinetics models that are frequently used to describe adsorption processes are pseudo-first-order model (Lagergren model) and the pseudo-second-order kinetics model (Ho and McKay model) [44,45,46]. The following equations are used to describe the kinetics models:

The pseudo-first-order kinetics equation (Lagergren model) [46]

$$\ln\left(q_{e}-q_{t}\right)=\ln q_{e}-k_{1}t$$

where q_e_ is the equilibrium adsorption capacity (µg g^−1^);

q_t_ is the adsorption capacity at a specific time—t (µg g^−1^);

k_1_ is the pseudo-first-order speed constant (min^−1^).

The pseudo-second-order kinetics equation (Ho and McKay model) [45]

$$\frac{t}{q_{t}}=\frac{1}{k_{2}q_{e}^{2}}+\frac{1}{q_{e}}$$

where q_e_ is the equilibrium absorption capacity (µg g^−1^);

q_t_ is the adsorption capacity at a specific time t (µg g^−1^);

k_2_ is the pseudo-second-order speed constant (g µg^−1^ min^−1^);

t is the contact time (min).

The pseudo-first-order speed constant (k_1_) and the adsorption capacity (q_e,calc_) are determined from the slope of the linear dependence ln(q_e_−q_t_) = f(t)—the linearised form of pseudo-first-order model—used during kinetics studies.

The slope of the linear dependence between t/q_t_ and t is used to evaluate the pseudo-second-order speed constant and the adsorption capacity (q_e,calc_) when the pseudo-second-order model is used during kinetics studies. By correlation of these computed kinetics parameters with the correlation coefficient R^2^, it is possible to establish which model best describes the investigated adsorption process. To consider that a model better describes the studied adsorption process, the correlation coefficient value needs to be closely related to 1. Temperature, pH and all reactions occurring during the adsorption process impact this correlation [47,48,49].

##### Intraparticle Diffusion

It is well established from the large number of experimental data that the adsorptive processes carried out on porous materials might be explained by a three stage mechanism:

(1) Diffusion of the ions through the film surrounding the adsorbent particles. In this case the process is driven by the concentration difference transversing the surrounding film [50,51];
(2) Intraparticle diffusion or internal diffusion consist of diffusion inside the adsorbent material pores. Such a process can be limited by pore size; in the case that the pores are not large enough, the adsorbed particles cannot escape from the adsorbent surface force field. Similarly, when the material pores are large enough, the adsorbed particles can easily escape from the adsorbent surface force field [50,52];
(3) Adsorptions/desorptions of adsorbate on adsorbent active sites are physical, physicochemical or chemical adsorptions [50,52,53].

To distinguish whether film diffusion or intraparticle diffusion is the rate-determining step, experimental data obtained from kinetics studies are fitted using the Weber and Morris model [54,55]:

$$q_{t}=k_{dif}\cdot t^{0.5}+C$$

where q_t_ is the adsorption capacity at t time, µg; g^−1^;

k_diff_ is the speed constant for intraparticle diffusion, µg g^−1^·min^−0.5^;

C is the constant correlated with the thickness of the liquid film surrounding the adsorbent particles.

The dependence between q_t_ and t^0.5^ must be a curve with the highest possible linearity passing through the origin (C = 0) when the intraparticle diffusion is the only factor that determines the studied process speed. Otherwise, the adsorption kinetics is influenced by both intraparticle and film diffusion processes.

##### Thermodynamic Studies

The activation energy associated with the Ga(III) adsorption process is used to determine whether the studied process is a chemical or a physical one [56]. The Arrhenius equation is used to calculate the activation energy value, and, in this case, the speed constant obtained from the kinetics model that better describes the adsorptive process is used:

$$\ln k_{2}=\ln A-\frac{E_{a}}{RT}$$

where k_2_ is the speed constant, g min^−1^ µg^−1^;

A is the Arrhenius constant, g∙min^−1^ µg^−1^;

E_a_ is the activated energy, kJ mol^−1^;

T is the absolute temperature, K;

R is the ideal gas constant, 8.314 J mol^−1^ K^−1^.

In practice, the slope of the linear dependence between k_2_ and 1/T is used to compute the activation energy value. Furthermore, the adsorption mechanism can be clarified by evaluating the value for ΔG^0^ by using the Gibbs–Helmholtz equation [57]:

$${∆G}^{0}={∆H}^{0}-T{∆S}^{0}$$

where ΔG^0^ is the free Gibbs energy standard variation (kJ mol^−1^);

ΔH^0^ is the enthalpy standard variation (kJ mol^−1^);

ΔS^0^ is the entropy standard variation (J mol^−1^ k^−1^);

T is the absolute temperature (K).

The evaluation of the values for ΔS^0^ and ΔH^0^ by using the van’t Hoff equation represents a prerequisite for further computation of the value of ΔG^0^. The slope of linear dependency between lnK_d_ and 1/T is used for the calculation of these two parameters.

$$\ln K_{d}=\frac{{∆S}^{0}}{R}-\frac{{∆H}^{0}}{RT}$$

where K_d_ is the equilibrium constant;

ΔS^0^ is the entropy standard variation (J mol^−1^ k^−1^);

ΔH^0^ is the enthalpy standard variation (kJ mol^−1^);

T is the absolute temperature (K).

R is the ideal gas constant (8.314 J mol^−1^ K^−1^).

The adsorption process equilibrium constant is calculated as the ratio of the adsorption capacity at equilibrium, q_e_, to the equilibrium concentration, C_e_.

$$K_{d}=\frac{q_{e}}{C_{e}}$$

The positive value of the standard enthalpy (ΔH°) represents the energy required to bring the adsorbate in contact with the adsorbent surface. The occurrence of different interactions between adsorbent and adsorbate (electrostatic or complex interactions) proves the adsorbent affinity for adsorbed particles. Based on the value computed for standard enthalpy, it can be stated that the studied process is either endothermic (ΔH° < 50 kJ mol^−1^, physical sorption) or an exothermic one (ΔH° > 50 kJ mol^−1^, chemisorption) [58]. Negative values of the Gibbs energy variation prove that the studied adsorptive process is spontaneous and natural. A positive value of the entropy change gives us the adsorption occurring at the adsorbent/solution interface.

It is well known that every molecule has a certain energy, which could be in the form of potential or kinetic energy. In this context, activation energy can be defined as the minimum kinetic energy that the reactants must have in order for chemical reactions to occur.

So, in the context of studied adsorptive processes, activation energy is the minimal energy required for adsorption at the solid–liquid interface. In order to understand the adsorption mechanism, the molecular forces governing the adsorptive process must be evaluated [58].

##### 2.2.6. Equilibrium Studies/Adsorption Isotherms

The equilibrium adsorption capacity represents an important parameter for analysis and optimal design for the adsorbent/adsorbate system [59]. Taking into account the experimental data obtained, adsorption isotherms provide information on the maximum adsorption capacity and the adsorption mechanism process.

###### Langmuir Isotherm

The Langmuir isotherm is substantiated by taking into account the following considerations: (i) the active centres on the surface of the solid adsorbent are at a constant number, and are identical and uniformly distributed on the surface; (ii) each active centre can adsorb a single molecule, so the adsorption layer must be strictly monomolecular, and the adsorption tends to limit correspondence to the occupation of all active centres on the surface; (iii) the heat of adsorption of the active centres is considered to be equal and independent of the degree of surface coverage, and no interactions occur between neighbouring molecules [60,61].

The nonlinear form of the Langmuir isotherm is described by [61]:

$$q_{e}=\frac{q_{L}K_{L}C_{e}}{1+K_{L}C_{e}}$$

where q_e_ is the maximum adsorption capacity (µg g^−1^);

C_e_ is the equilibrium concentration of metallic ion in solution (µg L^−1^);

q_L_ is the Langmuir maximum adsorption capacity (µg g^−1^);

K_L_ is the Langmuir constant.

R_L_ is a dimensionless constant representing the fundamental characteristic of the Langmuir isotherm, being known as the separation factor or the equilibrium parameter. The separation factor is evaluated by using the following equation:

$$R_{L}=\frac{1}{1+K_{L}C_{o}}$$

where R_L_ is the separation factor;

K_L_ is the Langmuir constant (L µg^−1^);

C_0_ is the initial concentration (µg L^−1^).

###### Freundlich Isotherm

The Freundlich isotherm is applicable when the adsorption process occurs on heterogeneous surfaces. The empirical isotherm equation describes the adsorbent material’s heterogeneous surface alongside the exponential distribution of active centres and their energy [62].

The nonlinear form of the isotherm is described by equation [63]:

$$q_{e}=K_{F}C_{e}^{1/n_{F}}$$

where q_e_ is the maximum adsorption capacity (µg g^−1^);

C_e_ is the equilibrium concentration of metallic ion in solution (µg g^−1^);

K_F_ și *n_F_* are the characteristic constants that can be related to the relative adsorption capacity of the adsorbent and the intensity of adsorption.

The value of n depicts the degree of nonlinearity between the concentration of the solution and the adsorption process, and it is as follows:

- If n = 1, then adsorption is linear;
- If n < 1, then adsorption is a chemical process;
- If n > 1, then adsorption is a physical process.

It is established that when n has values between 1 and 10, the adsorption process is a good one [64,65].

###### Sips Isotherm

The Sips isotherm is a combination of the Langmuir and Freundlich isotherm, defined in the nonlinear form by the following equation [66]:

$$q_{e}=\frac{q_{S}K_{S}C_{e}^{1/n_{S}}}{1+K_{S}C_{e}^{1/n_{S}}}$$

where q_S_ is the maximum adsorption capacity (µg g^−1^);

K_S_ is the constant related to the adsorption capacity of the adsorbent;

n_S_ is the heterogeneity factor.

The Sips isotherm can be reduced by the previous two isotherms; at a low adsorbent concentration, the adsorption process follows the Freundlich isotherm, whereas, at higher adsorbent concentration, the adsorption process follows the Langmuir isotherm [62]. Sips isotherm parameters are used to compute the separation factor, which is a dimensionless equilibrium parameter:

$$R_{S}=\frac{1}{1+K_{S}C_{0}^{1/n_{S}}}$$

where R_S_ is the separation factor;

K_S_ is the Sips constant;

n_S_ is the heterogeneity factor;

C_0_ is the initial concentration (µg L^−1^).

The value of the separation factor R_S_ allows for the evaluation of adsorption type, being an essential characteristic of the Sips isotherm. If R_S_ > 1, the adsorption process is unfavourable, resulting in a concave shape isotherm; if R_S_ = 1, the isotherm is linear; if 0 < R_S_ < 1, the isotherm has a convex shape, and the adsorption process is favourable; and if R_S_ = 0, adsorption is irreversible.

The isotherms are obtained by the graphical representation of the linearised equation q_e_ = f(C_e_), and the specific parameters of each isotherm used to model the experimental are computed from the slopes of its linear representation, respectively, from the ordinate at the origin.

## 3. Results and Discussion

### 3.1. Material Synthesis and Characterisation

#### 3.1.1. Fourier Transform Infrared Spectroscopy (FT-IR) Analysis

**Figure 2:**
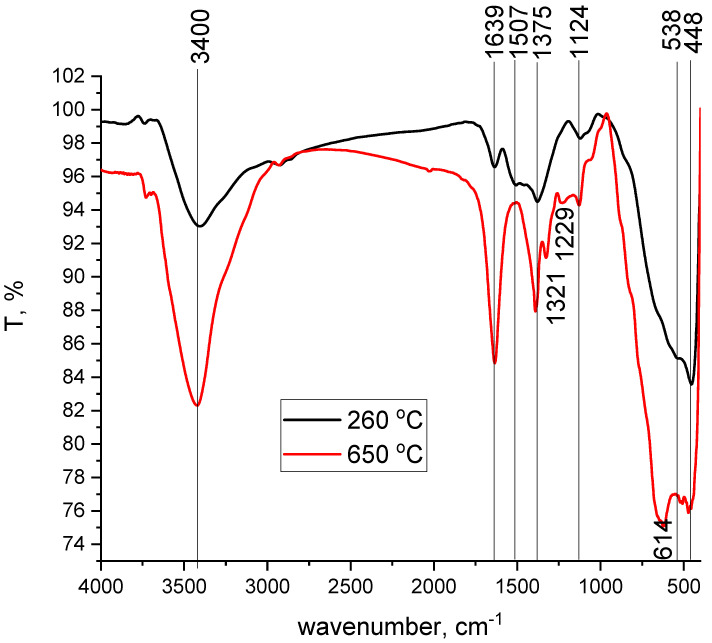
FT-IR spectra recorded for MgFe_2_O_4_ adsorbent material treated at 260 °C and 650 °C.

Figure 2 depicts the FT-IR spectra recorded for the MgFe_2_O_4_ adsorbent material thermally treated at 260 °C and 650 °C.

By comparing the spectra illustrated in Figure 2, we can observe that the treatment temperature leads to some changes in the specific bands for each material [38]. By analysing the data depicted in Figure 2, we can observe the presence of two peaks located at 3400 and 1639 cm^−1^, which are associated with –OH stretching and bending vibrations [38]. Further, we can observe that the main changes appear in the region 1500–1000 cm^−1^, in bands that are specific for the stretching vibration of the carbon groups. In the case of the adsorbent material obtained by thermal treatment at 650 °C, we can see the emergence of two new bands at 1321 and 1229 cm^−1^. The M-O bands are observed in the spectral range 1000–400 cm^−1^, and the increase in the treatment temperature leads to an intensification of these bands concomitant with the apparition of a new vibration at the wave number 614 cm^−1^, vibration specific to Fe-O bonds.

#### 3.1.2. Atomic Force Microscopy (AFM) Analysis

Based on the images recorded during AFM analysis, the values of the following parameters were computed: average roughness (Sa), mean square root roughness (Sq), maximum peak height (Sp), maximum valley depth (Sv), maximum peak-to valley height (Sy), surface kurtosis (Sku) and surface skewness (Ssk) (these values being depicted in Table 1).

The obtained results show the presence of roughness in both cases, but, when the treatment temperature increased to 650 °C, it led to an increase in the roughness of this sample. This difference between the samples was due to the change in morphology that is also visible in the recorded micrographs. In addition, the heights of the particular surface formations were measured, providing the authenticity of the information regarding the uniformity of the clusters and/or particles. These measured heights can be directly compared with the maximum peak height (Sp) values, since the measurements were performed on the formations that presented bigger heights (the lighter coloured formations in the AFM images).

**Figure 3:**
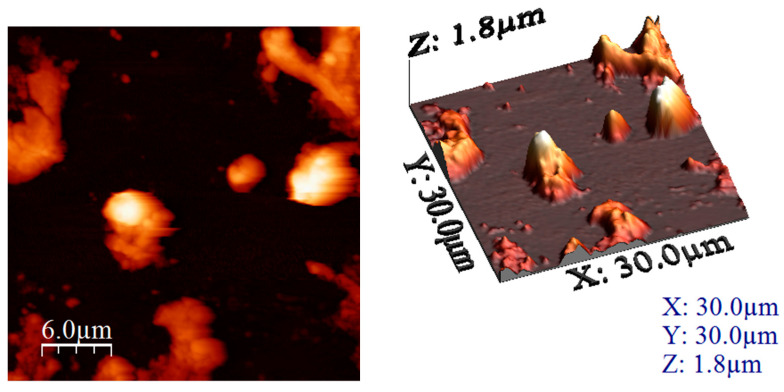
AFM images of MgFe_2_O_4_ heat treated at 260 °C (2D left and 3D right).

The measured heights are 700–800 nm and the sizes are between 5 and 6 μm. The sample formed a sort of film of several nm thickness around the clusters, which was visible from the height measurement (images presented in Figure 3).

**Figure 4:**
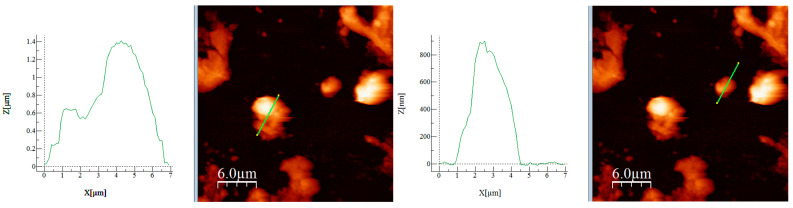
Calculated heights for the MgFe_2_O_4_ treated at 260 °C in the selected areas.

Magnesium exhibited uneven clusters of different widths and heights, as shown in images presented in Figure 4. The agglomerations had heights between 100 and 250 nm and sizes between 2 and 4 μm.

**Figure 5:**
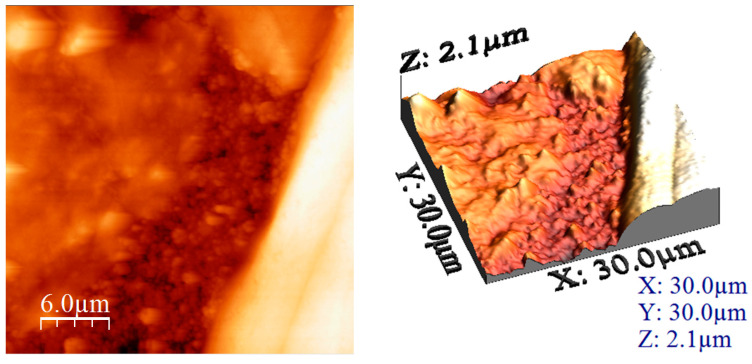
AFM images of MgFe_2_O_4_ treated at 650 °C (2D left and 3D right).

Further, the AFM images for the adsorbent material prepared by thermal treatment at 650° were recorded. In this case, by analysing the images given in Figure 5, we can notice that the studied adsorbent material suffered huge changes at this treatment temperature, showing the presence of a large number of joined wires.

**Figure 6:**
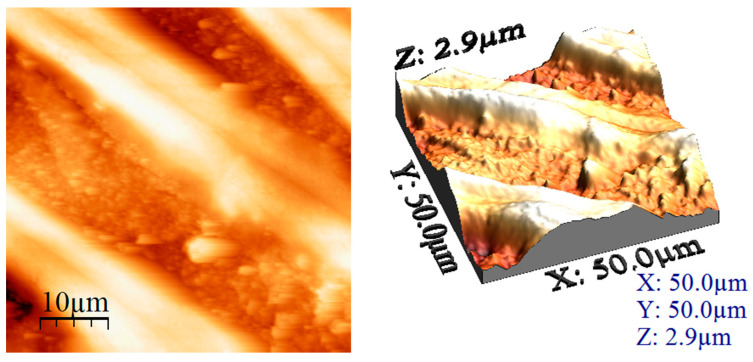
AFM images of MgFe_2_O_4_ treated at 650 °C (2D left and 3D right) covering larger area (50 × 50 μm).

Further, the AFM images for a larger surface (data depicted in Figure 6) were recorded. By analysing these images, we can conclude that the prepared adsorbent material consisted of multiple joined wires that had almost the same size and same distance between them.

**Figure 7:**
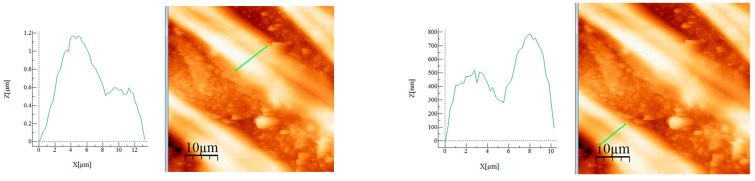
Calculated height for the MgFe_2_O_4_ treated at 650 °C (50 × 50 μm) in the selected areas.

Figure 7 depicts the AFM images used to calculate the heights for the prepared adsorbent material. The calculated size values were between 500 nm and 1.2 μm, whereas one of the wires was taller than the other one.

#### 3.1.3. Optical Microscopy (10 × 10) Analysis

**Figure 8:**
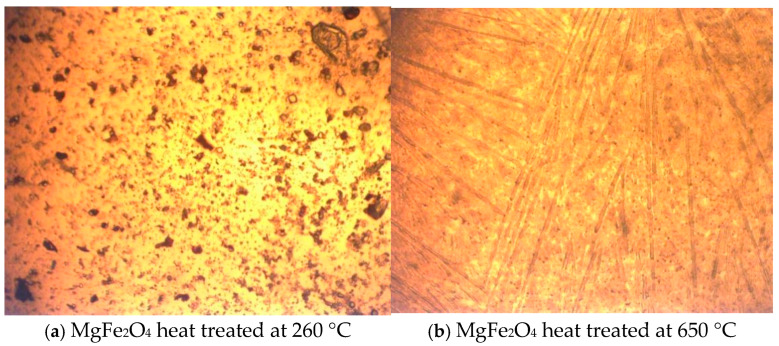
Optical microscopy images recorded for MgFe_2_O_4_ adsorbent material treated at 260 °C and 650 °C.

Optical microscopy images are used to evidence changes occurring in the morphology of adsorbent materials when they are treated at different temperatures. The images presented in Figure 8 demonstrate that the surface of the material obtained by thermal treatment at 260 °C is made up of round-shape granules, although the surface of the adsorbent material obtained by thermal treatment at 650 °C consists of linked wires. In this context, the magnesium wires are the most interesting ones, since they come in pairs, with two wires linked together. In addition, the distance between the paired wires seems to be very structured and almost repetitive (Figure 8a,b).

A very low adherence on the glass substrate was observed in the case of the adsorbent material prepared by thermal treatment at 260 °C, an adherence which increased in the sample prepared at a higher temperature (650 °C). Still, after several deposition attempts, the layer was not formed on the glass substrate.

#### 3.1.4. Magnetic Measurements

**Figure 9:**
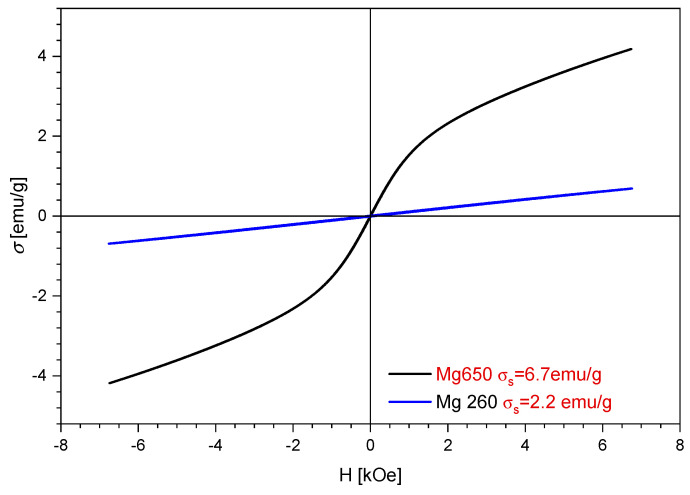
Magnetisation MgFe_2_O_4_ material heat-treated at 260 and 650 °C.

Figure 9 shows and compares the magnetisation curves of materials calcined at 260 °C and 650 °C.

By analysing the data presented in Figure 9, we may conclude that the prepared adsorbent materials exhibited superparamagnetic behaviour. Raw material calcination led to an increase in the saturation magnetisation from 2.2 emu g^−1^ in the case of the sample thermally treated at 260 °C to 6.7 emu g^−1^ for the sample thermally treated at 650 °C. In the particular situation of the sample thermally treated at 260 °C, we obtained a coercive field of 0.084 kOe and a remanent magnetisation of 0.009 emu g^−1^. When it came to the sample thermally treated at 650 °C, the decrease in the coercive field to 0.005 kOe and an increase in the remanent magnetisation with a value of 0.01 emu g^−1^ was observed.

### 3.2. Ga(III) Adsorption Experiments

#### 3.2.1. Solid/Liquid Ratio Effect

**Figure 10:**
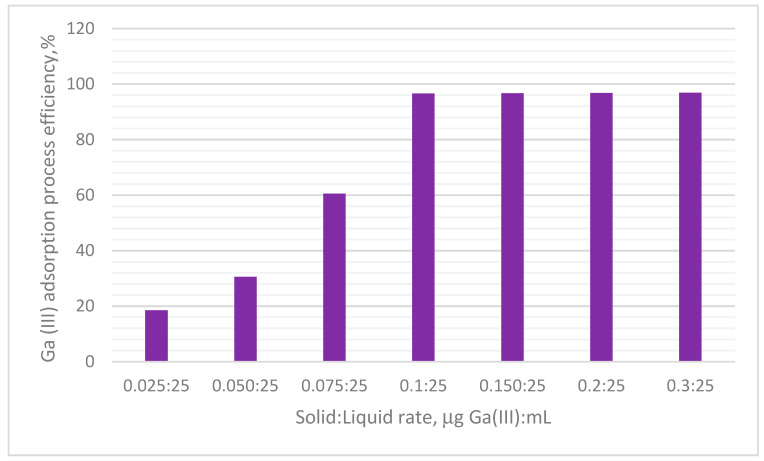
Solid/liquid rate effect.

Figure 10 presents the experimental data obtained regarding the effect of the S:L ratio on the efficiency of Ga(III) recovery by adsorption on the MgFe_2_O_4_ adsorbent material.

By analysing the experimental data depicted in Figure 10, it can be noticed that, when the S:L ratio increased, the degree of recovery of Ga(III) increased, up to the ratio S:L = 0.1 g:0.025 L, (~97% Ga(III) recovery degree), after which the degree of recovery remained constant. For this reason, the optimal S:L ratio for any further studies is S:L = 0.1 g:0.025L.

#### 3.2.2. Influence of the pH Value on Ga(III) Adsorption Process

**Figure 11:**
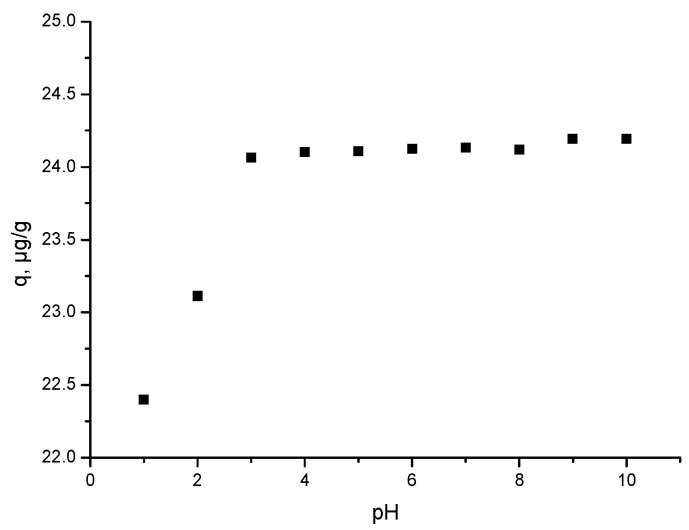
pH influence on the Ga(III) adsorption process.

In Figure 11, the experimental data that show the effect of the pH of the Ga (III) solution on the material adsorption capacity are depicted.

The initial pH of the Ga(III) solution represents one crucial parameter that determines the maximum efficiency of the adsorptive process. The pH value determines any further speciation of the metallic ions into the aqueous solution [67]. A chemical speciation diagram generated with Hydra/Medusa software and presented by Segala et al. [68] depicts that the precipitation of Ga(III) ions occurs at a pH higher than 3. According to Gondhalekar and Shukla [67], Ga(III) ions are found as hexa-hydrated ions ($Ga{\left(H_{2}O\right)}_{6}^{3+}$). These aqua ions undergo the hydrolysis process acting as acids. In this context, at a pH lower than 3, a higher number of H^+^ ions leads to a rivalry between Ga(III) and H^+^ ions for active adsorbent sites [67,68,69], thus limiting the Ga(III) ions adsorption. In this context, analysing the data presented in Figure 11 can conclude that the Ga(III) adsorption capacity increased with the pH increase, reaching a maximum value of ~24 µg Ga(III) g^−1^ MgFe_2_O_4_ for a pH of 3, this value being considered optimum for the studied process and to be used for any further experiments.

#### 3.2.3. Influence of Contact Time and Temperature

**Figure 12:**
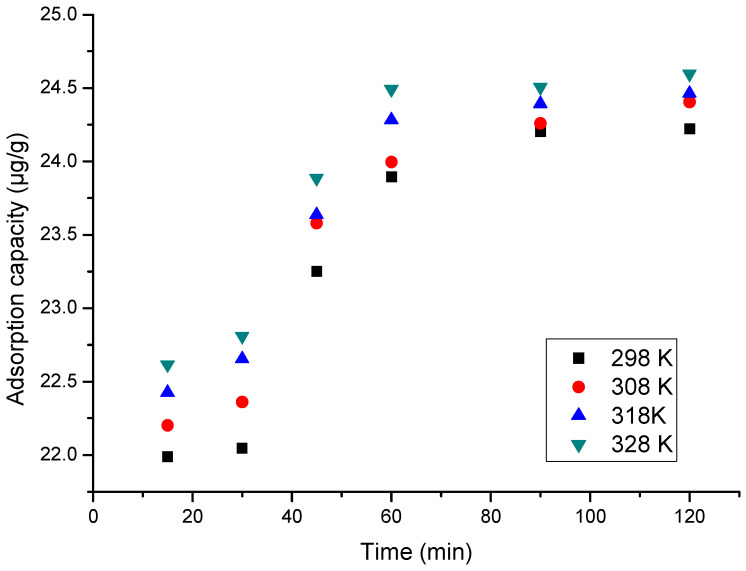
Contact time and temperature effect on material adsorption capacity.

The contact time effect was determined by varying it in a range between 15 and 120 min at four different temperatures (298 K, 308 K, 318 K and 328 K). The obtained experimental data are depicted in Figure 12.

From the experimental data depicted in Figure 12, we can observe that the material adsorption capacity increased with the increase in contact time. After 90 min, the adsorption capacity remained constant at approximately ~24 µg Ga (III) g^−1^. In addition, when the temperature increased, the adsorption capacity increased, but insignificantly; hence, any subsequent studies are to be carried out at 298 K.

#### 3.2.4. Kinetics Studies

**Figure 13:**
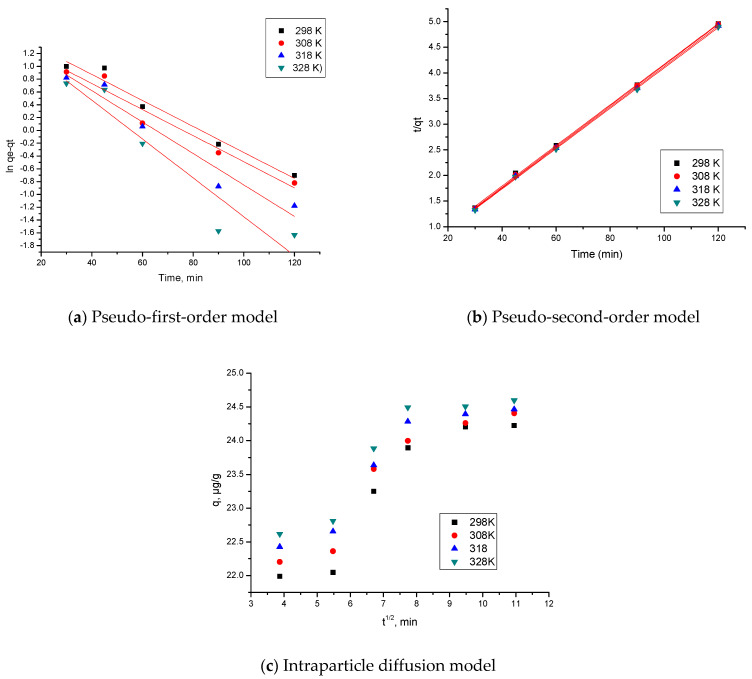
Performed kinetics studies.

To truly comprehend the Ga(III) recovery process, it is mandatory to study the adsorption kinetics process. In this context, the pseudo-first-order and pseudo-second-order kinetic models were used to model the obtained experimental data (results are depicted in Figure 13a,b).

In order to determine whether diffusion through the film or intraparticle diffusion was the rate-determining step, kinetics experimental data were further modelled according to the Weber and Morris model (Figure 13c).

Table 2 presents the values of the speed constants, the calculated adsorption capacity and the values obtained for the parameters K_diff_ and C obtained after the experimental data were modelled using the kinetics models. In addition, in the same table, the values of correlation coefficient, R^2,^ are presented.

The data reported in Table 2 prove that the Ga(III) adsorption process is effectively described by using the pseudo-second-order kinetic model. The value of the correlation coefficient (R^2^~1) supports this conclusion. Furthermore, q_e,calc_ based on the pseudo-second-order isotherm has a value comparable to the value experimentally obtained, q_e,exp_. Also, we can observe that the temperature presented a minor influence on the values of the parameters k_2_, q_e,calc_, so we consider that it is not necessary to work at temperatures higher than 298 K.

Due to the fact that the graphical representation of q_t_ versus t_1/2_ is not a straight line passing through the origin (in consonance with the Weber–Morris intraparticle diffusion model [70]), we can consider that the studied adsorption process is taking place in several stages. By analysing the data presented in Figure 13c, we can observe the presence of three linear parts in the IPD modelled data. The first stage is related to the mass transfer through the film that surrounded the adsorbent particles [70]. This stage took place for 30 min; when the adsorption rate speed decreased, then the second stage took place. This stage can be related to the diffusion of the Ga(III) ions to the adsorption sites located in the adsorbent internal pores. The adsorption equilibrium was reached after 90 min, leading to a constant adsorption capacity due to the limited number of free adsorption sites [70,71]. In light of all these findings, we can state that the Ga(III) adsorption on MgFe_2_O_4_ is influenced by intraparticle diffusion in addition to diffusion through the film. The data presented in Table 2 show that the K_diff_ value increased with the temperature increase. It is also noted that the diffusion constants specific to stage 1 were higher than the diffusion constants specific to stage 2, implying that stage 2 was the speed determinant stage [72].

#### 3.2.5. Activation Energy Determination

**Figure 14:**
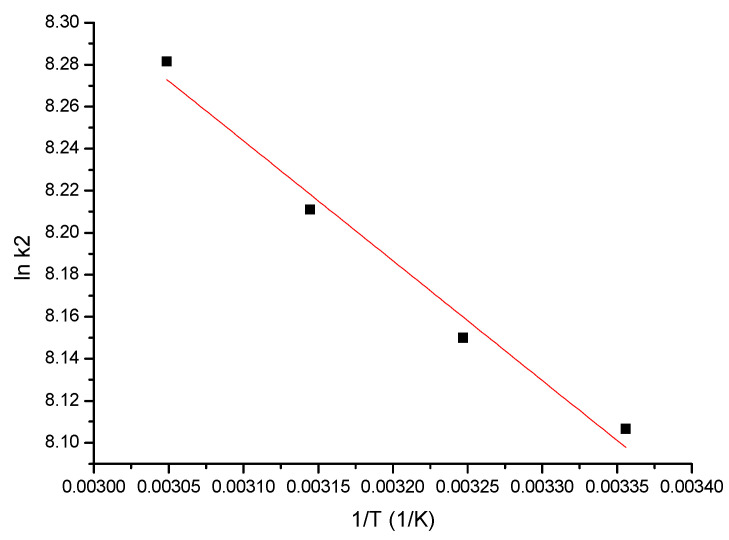
lnk_2_ vs. 1/T.

The activation energy (E_a_) value for the Ga (III) adsorption on the MgFe_2_O_4_ adsorbent material was computed by using the Arrhenius equation and the pseudo-second-order kinetics model constant rate, k_2_. The value of the activation energy revealed information about the nature of the adsorption process, proving whether it was a physical or chemical one. The E_a_ value was obtained from the linear dependence between lnK_2_ and 1/T (Figure 14).

From the data obtained, we can observe that the activation energy had a value of 4.2 kJ mol^−1^, which means that the studied adsorption process was a physical one [73].

#### 3.2.6. Equilibrium Studies

**Figure 15:**
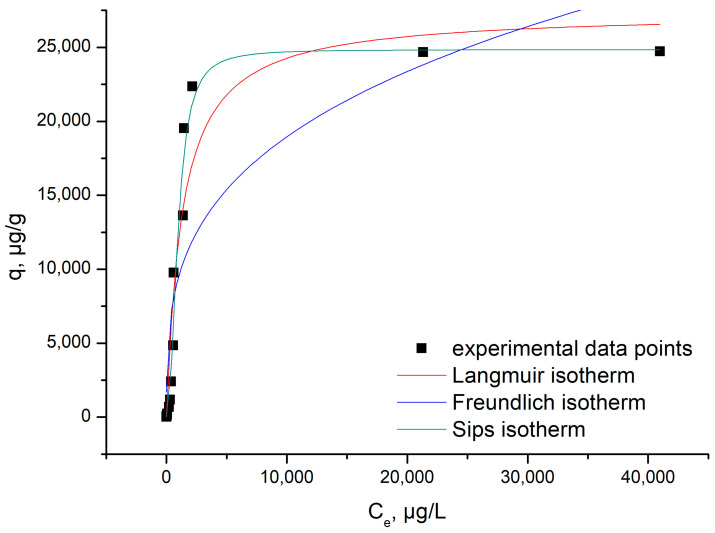
Equilibrium studies.

Adsorption mechanism can be decoded by modelling obtained experimental data using three different adsorption isotherms: Langmuir, Freundlich and Sips (obtained results are shown in Figure 15). By fitting the experimental data with the proposed adsorption isotherms, we were able to compute specific parameters, one of them being the maximum adsorption capacity of the studied adsorbent material.

Table 3 presents the specific parameters of each isotherm used to model the experimental data, being obtained from the slopes of the straight line and the ordinate from the origin.

The relationship between the equilibrium concentration of Ga (III) and the adsorption capacity proved that, as the equilibrium concentration increased, correspondingly, the adsorption capacity increased until equilibrium was reached, establishing the maximum experimentally obtained adsorption capacity, q_e,exp_ (~24.7 mg Ga(III) g^−1^).

From the data presented in Table 3, we can observe that the Sips isotherm best described the studied adsorption process, because the correlation coefficient, R^2^, was the closest to 1 (R^2^ = 0.9897), and the theoretical adsorption capacity was near the experimental one (~24.8 mg Ga(III) g^−1^).

#### 3.2.7. Thermodynamic Studies

**Figure 16:**
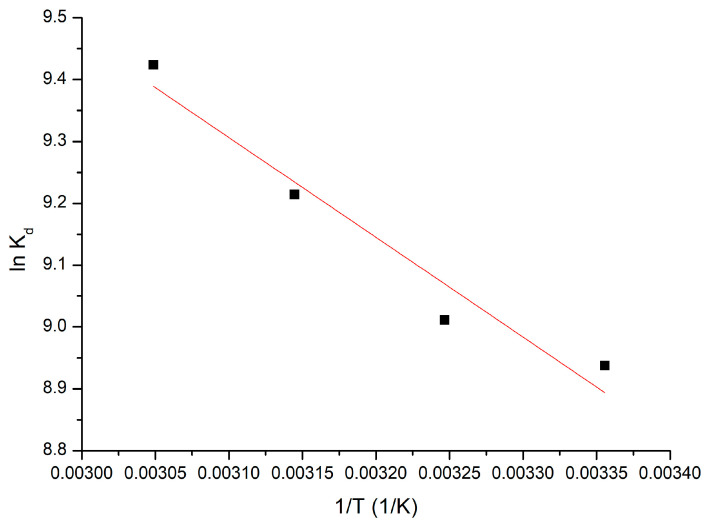
Thermodynamic studies.

Thermodynamic investigations were carried out at a temperature range between 298 and 328 K. The Gibbs free energy value was computed using the Gibbs–Helmholtz equation. The variations of standard entropy and standard enthalpy were obtained from the linear dependence between ln K_d_ and 1/T, as shown in Figure 16.

Table 4 presents the thermodynamic parameters calculated at the different temperatures used for this study.

From the positive value of ΔH^0^, we can affirm that the studied adsorption was endothermic. Similarly, the negative value of ΔG^0^ correlated with its increase in absolute value with the temperature increase, indicating that the adsorption process was spontaneous, being influenced by temperature. The fact that the ΔS^0^ value was positive indicates that the adsorption process was favoured, occurring at the adsorbent material–solution interface.

Over time, researchers have used different adsorbent materials for Ga recovery from different aqueous solutions. Such adsorbents are represented by mesoporous activated carbon [74], zeolite HY [17], TiO_2_ nanoparticles [75], bentonite [76], amidoxime resin [77], raw citrus peels [67] and polyacrylonitrile nanofibers loaded with di-(2-ethylhexyl) phosphoric acid [68]. Adsorbent materials used in the present study exhibited a maximum adsorption capacity of 24.7 mg Ga(III) per each gram of adsorbent, which is superior to the maximum adsorption capacity exhibited by other adsorbents (Table 5). Based on the data presented in Table 5, it can be observed that the newly prepared adsorbent materials present a good adsorptive capacity, being suitable candidates for any further industrial application.

## 4. Conclusions

The goal of the present work was to determine the optimal adsorption conditions for recovering gallium from aqueous solutions using a composite material based on iron and magnesium (MgFe_2_O_4_). This study concludes that a MgFe_2_O_4_ material can be used with good results to recover gallium ions from aqueous solutions by adsorption.

The MgFe_2_O_4_ composite material was synthesised by the coprecipitation method, thermally treated at two temperatures, 260 °C and 650 °C. The morphological and structural changes that appeared as a result of the thermal treatment were highlighted by Fourier transform infrared spectroscopy, atomic force microscopy and magnetic measurements. Taking into account that, as the temperature increases, the saturation magnetisation increases, we believe that the material can also be magnetically recovered after the appropriate thermal treatment.

The experimental results obtained at the laboratory scale demonstrated that the adsorbent material prepared presented an increased efficiency for Ga(III) ions recovery from aqueous solutions. Based on the obtained experimental data, we can conclude that the optimal conditions for Ga(III) adsorption are as follows: optimal S:L ratio = 0.1 g:0.025 L; optimum contact time is 90 min; initial concentration is 120 mg of Ga(III) L^−1^. By carrying out the experiments in these conditions, a maximum adsorption capacity of ~24.7 mg Ga(III) g^−1^ was obtained. It was proven that the temperature influenced the adsorption process, but, from an economic point of view, the studies were continued at a temperature of 298 K. In this context, it can be concluded that MgFe_2_O_4_ can be used as an adsorbent material for Ga(III) recovery from aqueous solutions.

## Figures and Tables

**Table 1:** Values of computed parameters obtained from AFM analysis.

Sample Name	Ironed Area (µm^2^)	Sa (µm)	Sq (µm)	Sp (µm)	Sv (µm)	Sy (µm)
MgFe_2_O_4_ 260 °C	950.344	0.177	0.266	1.756	−0.035	1.792
MgFe_2_O_4_ 650 °C	961.565	0.392	0.494	1.056	−1.056	2.113

**Table 2:** Kinetics parameters for the adsorption of Ga (III) onto MgFe_2_O_4_.

Pseudo-First-Order Model				
Temperature (K)	*q*_e,exp_ (µg g^−1^)	*k*_1_ (min^−1^)	*q*_e,calc_ (µg g^−1^)	R^2^
298	24.20	0.0203	4.425	0.9707
308	24.26	0.0204	4.70	0.9547
318	24.39	0.0246	4.99	0.9541
328	24.50	0.0304	5.71	0.9080
**Pseudo-Second-Order Model**				
**Temperature (K)**	***q*_e,exp_** **(** **µ** **g g^−1^)**	***k*_2_** **(g µg^−1^∙min^−1^)**	***q*_e,calc_** **(** **µ** **g g^−1^)**	**R^2^**
298	24.20	3316.12	25.25	0.9996
308	24.26	3463.32	25.18	0.9997
318	24.39	3681.34	25.31	0.9997
328	24.50	3950.35	25.44	0.9996
**Intraparticle Diffusion Model (IPD)**				
**Temperature (K)**	**K_diff_ (** **µ** **g·g^−1^ min^−1/2^)**	**C**		**R^2^**
298	0.31	20.54		0.8297
308	0.32	20.91		0.8440
318	0.34	21.25		0.8235
328	0.36	21.50		0.7848

**Table 3:** Parameters obtained from the modelling of experimental data with the adsorption isotherms.

Langmuir Isotherm			
*q*_m,exp_ (µg g^−1^)	*K*_L_ (L µg^−1^)	*q*_L_ (µg g^−1^)	*R* ^2^
24,731	7.7 × 10^−4^	27.384	0.9072
**Freundlich Isotherm**			
***K*_F_** **(µg g^−1^)**	**1/*n*_F_**		***R*** **^2^**
1178.07	0.30		0.9702
**Sips Isotherm**			
***K*** **_S_**	***q*_S_** **(** **µ** **g g^−1^)**	**1/*n*_S_**	***R*** **^2^**
4.3 × 10^−4^	24.851	0.05	0.9897

**Table 4:** Thermodynamic parameters for adsorption of Ga (III) onto MgFe_2_O_4_.

Δ*H*^0^ (J mol^−1^)	Δ*S*^0^ (J mol^−1^ K^−1^)	Δ*G*^0^ (kJ mol^−1^)				R^2^
13.42	61.54	298 K	308 K	318 K	328 K	0.9819
		−18.32	−18.94	−19.55	−20.17	

**Table 5:** Adsorptive capacity comparison for different adsorbent materials.

Adsorbent Material	Adsorption Capacity, mg g^−1^	Reference
mesoporous activated carbons	4.5	[74]
zeolite HY	7.9	[17]
TiO_2_ nanoparticles	8.0	[75]
bentonite	10.6	[76]
amidoxime resin (LSC700)	29.24	[77]
raw citrus peels, RCP	47.62	[67]
polyacrylonitrile nanofibers loaded with di-(2-ethylhexyl) phosphoric acid	33.13	[68]
MgFe_2_O_4_	24.7	Present paper

## Data Availability

The raw data supporting the conclusions of this article will be made available by the authors on request.
